# Association between parenting styles and dyslexia in primary school students: the mediating role of home literacy environment

**DOI:** 10.3389/fpsyg.2024.1382519

**Published:** 2024-06-13

**Authors:** Wanyi Wen, Xuanzhi Zhang, Kusheng Wu, Liwen Guan, Anyan Huang, Zhiya Liang, Xinle Yu, Qianfei Gu, Yanhong Huang

**Affiliations:** ^1^Mental Health Center of Shantou University, Shantou, Guangdong, China; ^2^School of Public Health, Shantou University, Shantou, Guangdong, China; ^3^Shantou University Medical College—Faculty of Medicine of University of Manitoba Joint Laboratory of Biological Psychiatry, Shantou, Guangdong, China; ^4^Department of Preventive Medicine, Shantou University Medical College, Shantou, Guangdong, China; ^5^Department of Health Care, Shantou Maternal and Child Health Hospital, Shantou, Guangdong, China

**Keywords:** dyslexia, parenting style, home literacy environment, reading ability, children

## Abstract

**Background:**

Despite an increasing amount of research on the relationship between parenting styles and neurodevelopmental disorders, there has been minimal focus on how parenting styles impact children’s reading abilities. The aim of this study was to investigate the potential mediating role of the home literacy environment in the connection between parenting styles and dyslexia.

**Methods:**

A total of 212 primary school students from grade 2–5 were recruited for this study. The Chinese Reading Ability Test was used to screen children with dyslexia. The home literacy environment was evaluated using a structured questionnaire that measured the frequency and quality of reading-related activities between parents and children. Egna Minnen Beträffande Uppfostran questionnaire was used to assess the parenting style, including emotional warmth, rejection, overprotection, and anxious rearing. It is a self-report tool filled out by the children themselves, used to assess their perceptions of their parents’ parenting styles. The structural equation modeling was carried out to evaluate the direct, indirect, and total effects of parenting styles on dyslexia.

**Results:**

Compared to control group, male children with dyslexia had lower scores in parenting styles characterized by emotional warmth, overprotecting and anxious rearing (*p* < 0.05), while female children with dyslexia only showed lower scores in anxious rearing (*p* < 0.05). Children with dyslexia lacked regular reading time (OR = 2.69, 95%CI: 1.04–6.97, *p* < 0.05), and have higher homework pressure compared to normal children (OR = 7.41, 95%CI: 1.45–37.82, *p* < 0.05). Additionally, emotional warmth, paternal overprotection and anxious rearing were negatively associated with dyslexia in children (all *p* < 0.05). Our findings indicate a strong correlation between dyslexia, home literacy environment, and parenting styles. In a structural equation model, the home literacy environment was identified as an independent mediator between parenting styles and dyslexia. The total effect of parenting styles on dyslexia is 0.55, with an indirect effect of 0.68 mediated by the home literacy environment.

**Conclusion:**

The findings of this study indicate that home literacy environment serves as a mediator between parenting styles and dyslexia in children. This study highlights how parenting styles influence dyslexia, offering key insights for aiding dyslexic children and guiding effective interventions.

## 1 Introduction

Developmental dyslexia (DD) is a neurodevelopmental disorder and is the most common learning disability in school-age children (Peterson and Pennington, 2015). It is characterized by difficulties in word recognition, spelling and decoding (Becker et al., 2017). Despite normal intelligence, complete sensory abilities, and adequate educational opportunities, children with dyslexia have serious and persistent problems in acquiring reading skills (Peterson and Pennington, 2015). This disorder is often linked to impairments in phonological processing, verbal processing speed, and verbal short-term memory (Snowling, 2012). Recent research has highlighted that dyslexia can influence various aspects of mathematical ability, such as understanding number concepts, memorizing arithmetic, and solving mathematical problems (Joyner and Wagner, 2020). This suggesting that difficulties in arithmetic, reading or spelling, are notably more prevalent among children with academic challenges (Georgitsi et al., 2021). Moreover, studies have demonstrated that DD affects approximately 10% of children (Doust et al., 2022) and 3–12.6% of school-age children in China (Feng et al., 2022), which can persist into adulthood. Unfortunately, dyslexia not only prevents children from enjoying reading, but also increases their learning difficulties and often deprives them of opportunities to develop other potential, which in turn has a considerable negative impact on mental health outcomes and behaviors (Soriano-Ferrer et al., 2021; Xiao et al., 2023). Therefore, DD is an important public health issue that deserves attention.

The etiology of dyslexia is multifactorial, with research indicating that it arises from a combination of genetic and environmental risk factors and their interactions (Peterson and Pennington, 2015). Dyslexia often recurs in families, with the majority of twin studies reporting a heritability of 40–60% (Gialluisi et al., 2021). Building on this foundation, recent genetic research has identified several genetic loci associated with dyslexia and proposed some candidate genes (Doust et al., 2022). These candidate genes, including *DCDC2*, *KIAA0319* (6p22.3), *DYX1C1*, *GCFC2*, *ROBO1* (3p12.3-p12.3), and *LOC388780*, most of which are not only linked to dyslexia but also to persistent individual differences in related cognitive skills (such as word reading, spelling, etc.) (Gialluisi et al., 2021; Doust et al., 2022). Furthermore, the identified candidate risk genes associated with neural migration and brain development indicate that dyslexia may stem from atypical neural migration during brain development (Gabrieli, 2009). Moreover, functional neuroimaging studies have revealed differences in brain function and connectivity that are characteristic of dyslexia (Perdue et al., 2022). While extensive research into the neurophysiological and genetic underpinnings has been instrumental in identifying the occurrence of dyslexia, it has provided limited guidance for its prevention and intervention. Dyslexia may be a lifelong condition, but timely and early intervention can improve reading abilities, thereby enhancing prognosis. As research progresses, scholars are delving deeper into environmental factors that heighten the risk of dyslexia, including prenatal and perinatal environments, school settings and family environments (Feng et al., 2022; Torppa et al., 2022). Among these, the family environment, closely related to children’s lives, plays a significant role in influencing dyslexia and is the most amenable to intervention. Its impact on identifying the causes of dyslexia and on early intervention studies is profound, necessitating further research into related aspects of dyslexia in the Chinese language.

Considering that families share both genetic material and environmental factors, it’s conceivable that dyslexia might also arise as a consequence of environmental influences linked to low literacy levels (Torppa et al., 2022). Many studies have shown that the occurrence of dyslexia is closely related to family environment (Dilnot et al., 2017; Huang et al., 2021). The home literacy environment (HLE) is used to describe the interactions, resources, and attitudes that children experience at home related to literacy, such as parent–child literacy-related activities, family books, usage of electronic devices etc. (He et al., 2014). Previous studies have studied the HLE from various aspects. Meanwhile, extensive research has underscored the relationship between the HLE and children’s reading development, acknowledging that while certain aspects of HLE, including quality interactions and access to reading materials, positively contribute to reading skills (Lau and Richards, 2020; Torppa et al., 2022), careful consideration is required regarding the role of screen time, which may have varied impacts. Recent American Speech-Language-Hearing Association (ASHA) recommendations suggest that increased screen time, instead, delays child brain and language development, aligning with the results of a cross-sectional study in the United States (Hutton et al., 2020). The HLE explains 12–18.5 percent of the variation in children’s language skills (Niklas et al., 2020). Frank Niklas et al. stated that the relationship between parental attitudes toward shared reading and children’s language ability was mediated by the HLE (Niklas et al., 2020). A Finnish longitudinal study also found that the HLE plays a crucial role in the development of dyslexia in children (Torppa et al., 2022). Importantly, a good reading atmosphere at home was a conducive condition for promoting the development of children’s early reading skills (Hamilton et al., 2016). Rashid conducted a study among dyslexic children and found a significant correlation between HLE and children’s passage comprehension as well as spelling scores (Rashid et al., 2005). Similarly, recent research has confirmed that Chinese children with dyslexia often come from homes with poor literacy environments (He et al., 2014; Feng et al., 2022). Therefore, the HLE is a crucial factor in the development of reading ability in children.

Parenting styles are the methods and forms commonly used by parents in the raising and education of their children, with a relatively stable style of behavior (Guo et al., 2022). Parenting styles permeate the parent–child interaction process and have a significant impact on children’s personality, mental health, and especially their academic performance (Watson et al., 2023; Zhong et al., 2023). Parenting styles are categorized into four types: emotional warmth, rejection, overprotecting and anxious rearing. Each style has distinct characteristics and impacts on children. Notably, adverse parenting styles, such as those marked by rejection or overprotection, are linked to an increased risk of emotional and behavioral problems among children (Zou et al., 2020). In contrast, an environment nurtured by parental support and engagement significantly diminishes these challenges, fostering a more positive developmental outcome. Conversely, a hostile or overly controlling parenting approach exacerbates children’s behavioral and emotional problems (Wang et al., 2024). Specifically, a parenting style characterized by high rejection and low emotional warmth may lead to problematic child-rearing practices, including insufficient supervision, harsh discipline, and a negative family environment (Penelo et al., 2010). A study conducted in Turkey indicated that parenting styles marked by rejection, lack of emotional warmth and overprotection predicted behavioral problems in children (Eruyar et al., 2020). Additional research indicates parenting styles characterized by overprotection and anxious rearing may increase the risk of dyslexia in children (Huang et al., 2021). This correlation is further highlighted by the challenges faced by parents of dyslexic children, emphasizing the difficulty in providing effective support and the significant impact of parenting styles on dyslexia (Natale et al., 2008; Carotenuto et al., 2017). On the other side, numerous studies have demonstrated that parenting care is necessary to meet the needs of children’s brain development and that positive parenting (authoritative parenting style) reduces the incidence of neurodevelopmental disorders in children (Chronis et al., 2007; Daelmans et al., 2017). This style is characterized by a warm, nurturing approach that combines clear expectations and guidance with respect for children’s autonomy, consistent with emotional warmth. Furthermore, a survey among Portuguese children has shown that positive parenting styles significantly contribute to higher reading processes, encompassing both syntactic and semantic aspects (Carreteiro et al., 2016). Therefore, parenting styles are particularly crucial for children with dyslexia, as these variables can either increase risk or serve as protective factors. Given the intricate relationship between parenting styles and children’s developmental outcomes, further research is essential to expand our understanding and provide actionable insights for fostering environments that support healthy development, particularly for children facing learning challenges such as dyslexia.

Despite the growing body of research on parenting styles and their impact on neurodevelopmental disorders, there has been limited focus on how parenting styles specifically influence dyslexic children. Therefore, further exploration of the mediating factors in the relationship between parenting styles and dyslexia in children is necessary. We propose that the HLE could be a significant predictor of dyslexia, potentially interacting with parent–child interaction at home and influencing children’s literacy development. To delve deeper into this relationship and its underlying mechanisms, selecting the appropriate assessment tool is crucial. It is with this necessity in mind that we chose the Egna Minnen Beträffande Uppfostran for Children (EMBU-C) questionnaire for our study. Our choice of the EMBU-C was driven by its comprehensive approach to evaluating parenting styles, which is critical for understanding the family environmental factors influencing dyslexia. Our preference for the EMBU-C over other instruments was based on several considerations. First, its detailed focus on children’s perspectives aligns with our research objective to explore the mediating role of the home literacy environment in the association between parenting styles and dyslexia. Secondly, the EMBU-C’s success in cross-cultural research guarantees its applicability to a wide audience. Its use in previous studies has proven effective in connecting parenting styles with outcomes like academic achievement and mental health issues (Saleem et al., 2015; Eruyar et al., 2020; Huang et al., 2021). Lastly, the questionnaire’s comprehensive nature allows for a deeper understanding of the complex interactions within the family that may contribute to or mitigate the risk of dyslexia.

To date, no prior studies have specifically examined the relationships among parenting styles, HLE, and dyslexia in Chinese children. Therefore, the aim of this study is to address two key questions: (a) Is there a correlation between dyslexia and both parenting styles and HLE among Chinese children; (b) Could the HLE serve as a mediator in the relationship between parenting styles and dyslexia.

## 2 Methods

### 2.1 Study populations

In this study, 53 children with dyslexia diagnosed were selected as the case group in the Mental Health Center, Shantou University Medical College. We used a random sampling method to select typically developing students from grades 2–5 at a public primary school as the control group in Shantou, China. The case and control groups were matched 1:3 according to gender, age and grade. After clarifying the objective of the research, a total of 212 children and their parents (53 in the case group and 159 in the control group) voluntarily agreed to participate in the study following informed consent.

The inclusion criteria of the dyslexia case group were consistent with the previous studies of our group (Huang et al., 2021). In brief, the screening criteria for children with dyslexia were as follows: (1) Intelligence Quotient (IQ) above 85 by the Combined Raven’s Test; (2) the scores on the Dyslexia Checklist for Chinese Children (DCCC) were two standard deviations higher than the average score of children in the same grade (Cronbach’s α = 0.97) (Hou et al., 2018); (3) at least 1 standard deviation below the average level of their actual grade on the Chinese Vocabulary Test and Assessment Scale (Cronbach’s α = 0.75) (Huang et al., 2020); (4) The Chinese language test score was below the 20th percentile among all students in the same grade; (5) The final diagnosis was made by a child psychiatrist based on the results of the Chinese Reading Ability Test (CRAT) (Cronbach’s α = 0.75) (Huang et al., 2020) and the fifth edition of Diagnostic and Statistical Manual of Mental Disorders (DSM-5) (Ma, 2021). Children with brain disease, traumatic brain injury, epilepsy, visual and auditory dysfunction and other neurodevelopmental disorders were excluded from the study. This study conformed to the ethical guidelines of the Declaration of Helsinki and was approved by the Ethics Committee of Mental Health Center, Shantou University Medical College, with the approval number SUMC-202036. The informed consent was obtained from all participants before the investigation.

### 2.2 Procedure

After the trained investigators clearly explained the purpose of the study and obtained informed consent from all participants, questionnaires were administered to both the children and parents in the case and control groups. The case group survey was conducted at the Shantou University Mental Health Center’s dyslexia specialist clinic, utilizing a one-on-one on-site questionnaire approach where each investigator individually guided a child with dyslexia through the EMBU-C and CRAT questionnaires. For the control group children, the questionnaire survey was carried out collectively within the classroom. During the survey phase, we received support from the class teachers, and the investigators monitored the process of completing the questionnaires. For questionnaires with missing responses, investigators will remind participants to complete the missing information. All researchers involved in administering the questionnaire underwent training prior to the study, covering the purpose and content of the survey, standard procedures for filling out the questionnaire, and strategies for resolving common issues. In addition, parents or other guardians were asked to complete questionnaires on demographic information, home literacy environment, and screening for dyslexia. Investigators uniformly collected all questionnaires. After Research Topic, they reviewed the completion status of the questionnaires, and for those that did not meet the requirements, such as missing items or illogical response options, parents were contacted to refill the questionnaire, which was then recollected. The quality control process of students filling in the questionnaire in the control group was consistent with that in the case group.

### 2.3 Measuring tools

#### 2.3.1 Egna Minnen Beträffande Uppfostran for Children

The EMBU-C is a powerful and objective tool which is used to evaluate the current relationship between parenting styles and children’s mental health. It is a self-report tool used to assess children’s perceptions of their parents’ parenting styles, filled out by the children themselves. Essentially, the use of questionnaires to understand children’s perspectives is valuable for exploring how various aspects of home life. The Chinese version (EMBU-C) for school-age children and adolescents, as translated and revised by Liming Tie et al., was applied in our study (Liming, 2018). The EMBU-C includes four dimensions, namely emotional warmth (EW), rejection (R), overprotecting (O), and anxious rearing (AR), made up of 10 items, respectively. The participants were asked to respond twice to each question item, evaluating the current parenting styles of their fathers and mothers, respectively. The items were rated on a four-point Likert scale from never (1 point) to always (4 point) where the higher the score on a dimension, the greater the tendency to represent parenting styles on that dimension. The Cronbach’s alpha of the EMBU-C was 0.82, and test–retest reliability was between 0.72 and 0.90. Statistical validation of the EMBU-C questionnaire has shown possess high reliability and validity across diverse cultural contexts, making it an appropriate tool for our research.

#### 2.3.2 Home literacy environment

Based on the HLE scale designed by He et al. (2014), this study made some changes to the items. Ten variables reflected the home literacy environment, such as Literacy-related activities, restrictions on using electronic devices, usage of electronic devices, shared TV, children’s learning habits, completion of homework by children, whether the child participated in extracurricular activities, frequency of encouraging children to participate activities and hours of outdoor. Children’s literacy-related behaviors, such as regular reading time, were measured according to the HLE variables assignment table. The higher the frequency of the behavior, the higher the score. The cumulative score of each item ranged from 0 to 24 among all the students (see the details in Supplementary Table S1).

#### 2.3.3 The Chinese Reading Ability Test

The Chinese Reading Ability Test (CRAT) was used to diagnose Chinese dyslexic children (Lin et al., 2020). The CRAT is applicable across all Simplified Chinese character regions. It is composed of five subscales, respectively the phonological awareness (PA), morphological awareness (MA), rapid automatized naming (RAN), orthographic awareness (OA), and reading ability (RA). The PA consists of three tests: Rhyme Recognition, Onset Recognition and Tone Recognition. The MA asks the children to combine each character in the first column with a character in the second column to form a meaningful compound word, and record the completion time and score. In the RAN, children are asked to name of all numbers twice, from left to right, from top to bottom, as quickly and accurately as possible. The OA was used to measure children’s knowledge of the structure of Chinese characters and their sensitivity to differentiate between Chinese and non-Chinese characters. The RA includes oral and written questions, as well as reading time. The higher the score and the shorter the time taken, the better the children have mastered the skill. The Cronbach’s alpha coefficient for the scale was 0.75 (Huang et al., 2020).

### 2.4 Statistical analysis

The statistical analyses were performed using IBM SPSS 26.0 Statistics for Windows (IBM Corp., Armonk, NY, United States) and R 4.2.3. (R Core Team, Vienna, Austria). The continuous variables were expressed using mean ± standard deviation (mean ± SD), and the categorical variables were expressed using frequencies and percentages. The individual sample t-test and chi-square test were used for group comparisons. Preliminarily, Spearman correlations were performed to examine the relationships among the home literacy variable, parenting styles and dyslexia to assess the multicollinearity of the variables (Xiao et al., 2022). Given the observed correlations, causality was examined using multiple logistic regression. The examination of the causality of the variables is necessary to carry out the mediation model (Bigozzi et al., 2023).

Then, based on the hypothesis, we used Structural Equation Modeling (SEM) to examine the mediation effects. We examined this association controlling for various child and family characteristics. After analyzing the missing data for patterns, we estimated missing data using the full information maximum likelihood option (MLR estimator) in M-plus version 8. For the indirect effects, we used a bootstrap resamples of 5,000 in order to obtain nonbiased 95% confidence intervals (CI) (Hayes, 2017). If the 95% CI did not contain zero, it indicated a significant mediating effect. All predictor variables were standardized to minimize multicollinearity. Besides, to examine the model fit, several fit indices were considered: Comparative Fit Index (CFI), Tucker–Lewis Index (TLI), Root Mean Square Error of Approximation (RMSEA), and Standardized Root Mean Square Residual (SRMR). The values of >0.90 for CFI and TLI, < 0.08 for RMSEA and < 0.05 for SRMR represent a good fit (Klumparendt et al., 2019). The significant level was set to 0.05.

## 3 Results

### 3.1 General characteristics of the participants

Table 1 displays the sociodemographic details of 53 dyslexic children and 159 controls. Concerning district, 43 (81.1%) children in the dyslexic group resided in urban areas, compared to 149 (93.1%) in the control group (*p* = 0.007). A significant difference was also observed in monthly family income between the two groups (*p* = 0.017). Compared to controls, the proportion of single-parent families (9.4% vs. 1.9%, *p* = 0.037) and family history of dyslexia (7.5% vs. 0.6%, *p* = 0.009) were much higher in dyslexic children. In terms of paternity, children with dyslexia were more likely to feel ignored and scolded by their parents (all *p* < 0.05). No significant differences were observed in sex, age, grade distribution, learning training before age 3, parental education levels or occupation of parents between the two groups (*p* > 0.05).

**Table 1:** General characteristics of the participants.

Variable	Overall (*n* = 212)	Dyslexic (*n* = 53)	Control (*n* = 159)	*P*
Sex, n (%)				1.000
Boys	152(71.7)	38(71.7)	114(71.7)	
Girls	60(28.3)	15(28.3)	45(28.3)	
Age (Mean ± SD)	9.13 ± 1.34	9.13 ± 1.35	9.13 ± 1.34	1.000
Grade				1.000
2	72(34.0)	18(34.0)	54(34.0)	
3	64(30.2)	16(30.2)	48(30.2)	
4	48(22.6)	12(22.6)	36(22.6)	
5	29(13.2)	7(13.2)	21(13.2)	
Single parent family				0.037
Yes	8(4.0)	5(9.4)	3(1.9)	
No	204(96.0)	48(90.6)	156(98.1)	
Place of residence				0.007
Rural	20(9.4)	10(18.9)	10(6.3)	
City	192(90.6)	43(81.1)	149(93.7)	
Family history of dyslexia				0.009
Yes	5(2.4)	4(7.5)	1(0.6)	
No	207(97.6)	49(92.5)	158(99.3)	
Learning training before age 3				0.628
Yes	86(40.6)	20(37.7)	66(41.5)	
No	126(59.4)	33(62.3)	93(58.5)	
Father’s educational level				0.127
Junior high school or below	51(24.1)	18(34.0)	33(20.8)	
High school or equivalent	67(31.6)	13(24.5)	54(34.0)	
Bachelor’s degree or above	94(44.3)	22(41.5)	72(45.3)	
Mother’s educational level				0.237
Junior high school or below	70(33.0)	15(28.3)	55(34.6)	
High school or equivalent	47(22.2)	9(17.0)	38(23.9)	
Bachelor’s degree or above	95(44.8)	29(54.7)	66(41.5)	
Occupation of father				0.136
Worker	25(11.8)	8(6.3)	17(10.7)	
Merchant	109(51.4)	24(45.3)	85(53.5)	
Organizational staff	33(15.6)	5(9.4)	28(17.6)	
Other	45(21.2)	16(30.2)	29(18.2)	
Occupation of mother				0.670
Worker	16(7.5)	6(11.3)	10(6.3)	
Merchant	87(41.0)	20(37.7)	67(42.1)	
Organizational staff	19(9.0)	5(9.4)	14(8.8)	
Other	90(42.5)	22(41.5)	68(42.8)	
Monthly family income				0.017
<5,000	47(22.2)	17(32.1)	30(18.9)	
~10,000	75(35.4)	22(41.5)	53(33.3)	
>10,000	90(42.5)	14(26.4)	76(47.8)	
Be scolded by parents				0.005
Yes	156(73.6)	48(81.6)	108(67.9)	
No	56(26.4)	5(9.4)	51(32.1)	
Ignore children’s feelings				0.035
Frequently	7(3.3)	3(5.7)	4(2.7)	
General	114(53.8)	35(66.0)	79(49.7)	
None	91(42.9)	15(23.4)	76(47.8)	

### 3.2 Comparison of parenting styles and CRAT between dyslexic and non-dyslexic children

**Figure 1:**
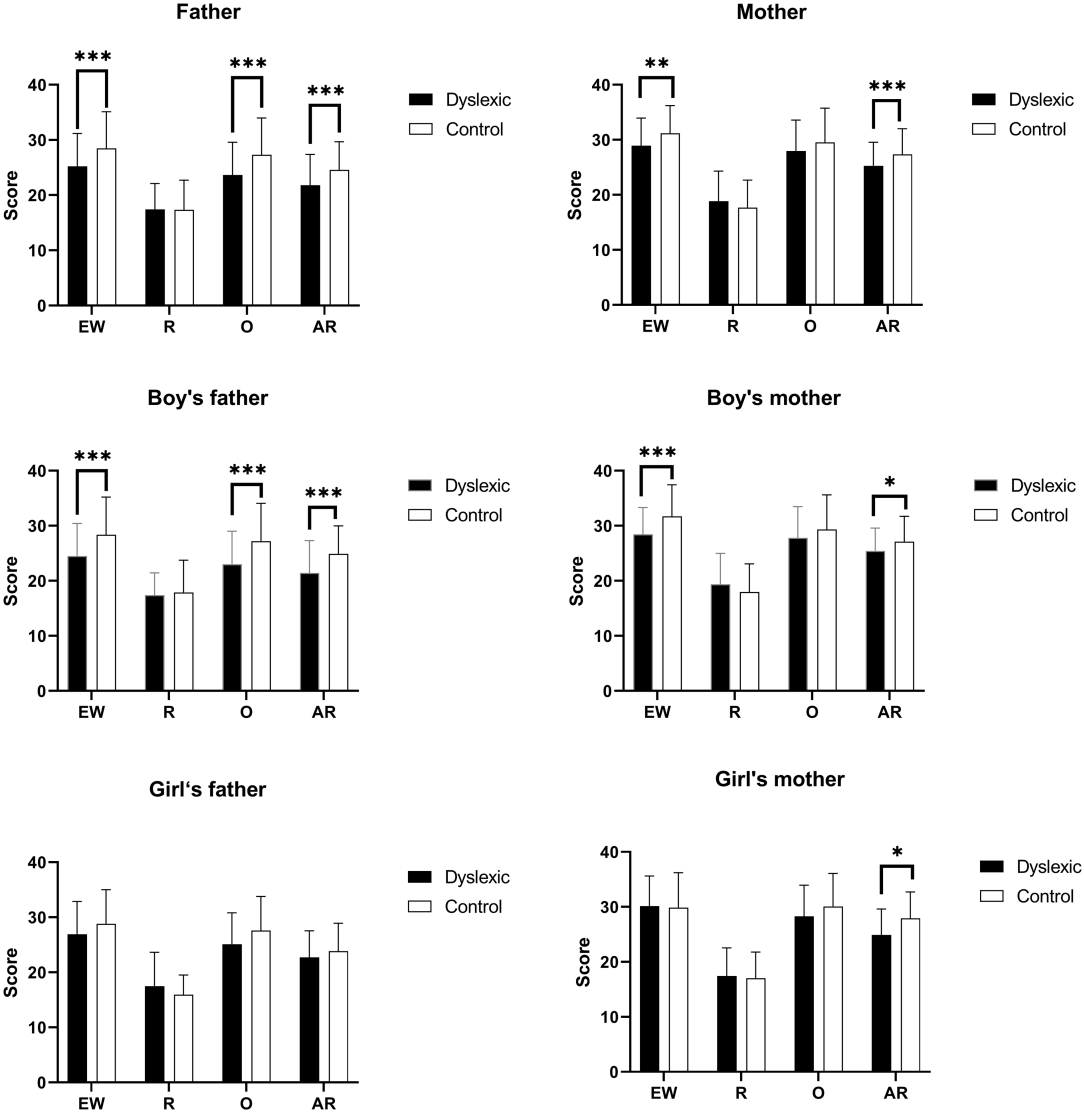
Comparison of parenting style scores between dyslexic group and control group across total and genders. EW, emotional warmth; R, rejection; O, overprotecting; AR, anxious rearing. *p < 0.05, **p < 0.01, ***p < 0.001. This part of the data analysis excluded children from single-parent families.

The scores for the different types of parenting styles of dyslexic and control children were shown in Figure 1. The scores for emotional warmth and anxious rearing of parents in the dyslexic group were lower than those in the control group (*p* < 0.01), and the scores for overprotecting of fathers were also significantly lower than those in the control group (*p* < 0.001). Based on gender stratification analysis, the results showed that there were differences in the scores of parenting styles between dyslexic boys and normal boys, mainly in the five dimensions of emotional warmth, anxious rearing and overprotection of the father, and emotional warmth and anxious rearing of the mother. Male dyslexic children scored significantly lower than controls on all of these factors (*p* < 0.05). However, among the maternal rearing style of girls, only anxious rearing had a statistically significant difference between the two groups (*p* < 0.05), with the dyslexic group scoring lower than the controls (*p* < 0.05).

Regarding CRAT, there were significant differences in the five dimensions between the two groups (all *p* < 0.05). The case group scored lower than the children in the control group and took longer time to complete the test on all subscales (Supplementary Table S2).

### 3.3 Correlation matrixes between the investigated factors

**Figure 2:**
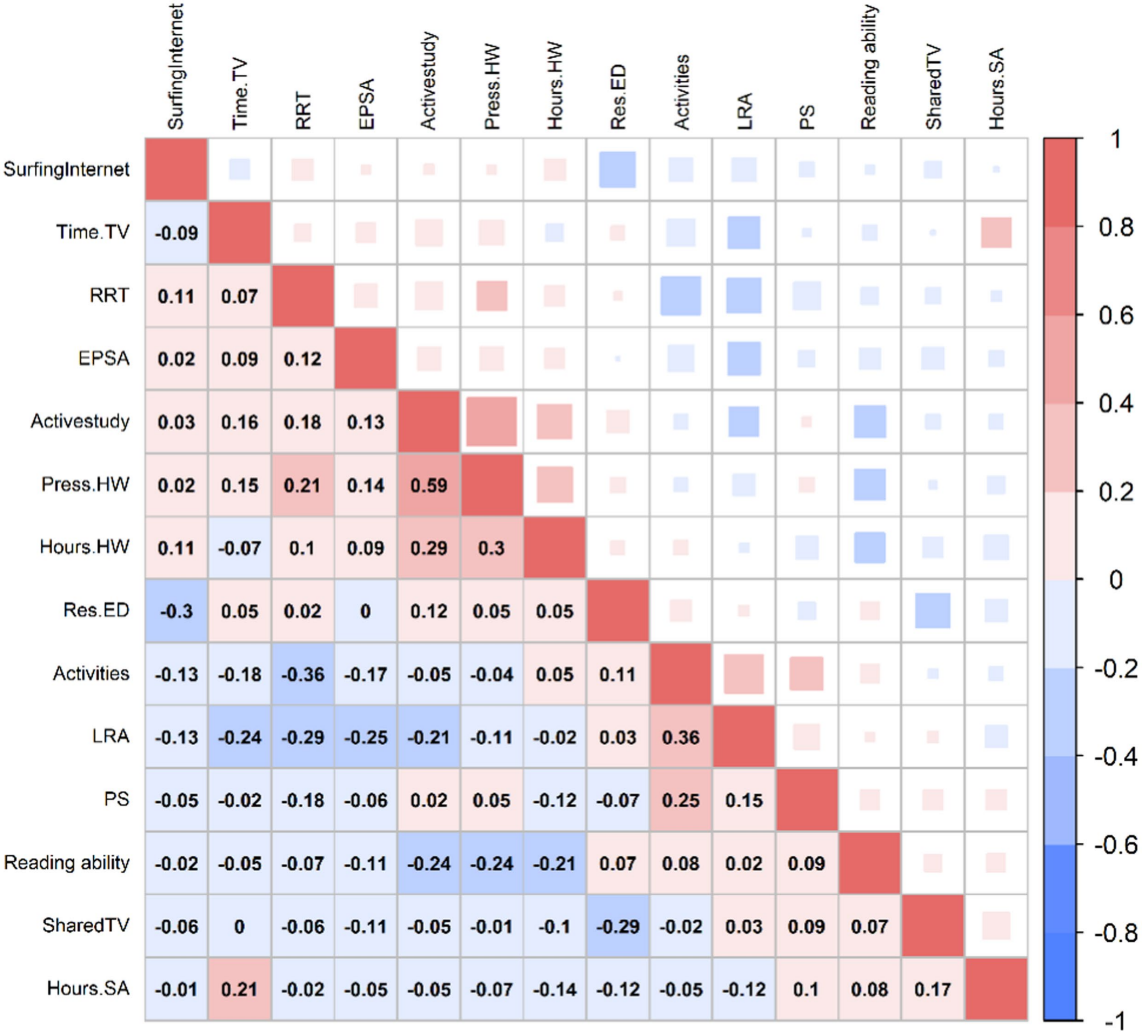
Correlation analysis matrix between variables. Reading ability, the total score of CRAT; PS, parenting styles; LRA, literacy-related activities; RRT, regular reading time; Activities, whether child participates in extracurricular activity such as reading; EPSA, whether parents encourage children to participate in sports activities; Press.HW, homework pressure; Hour.HW, hours of finishing homework; Time.TV, time spending on TV everyday; Hours.SA, hours of sports activities; Res.ED, restrictions on electronic devices; Shared TV, whether parents watching TV with children; Surfing Internet, whether the child is online.

Data were assessed for normality, outliers and multicollinearity prior to path analysis. No evidence of significant deviation from normality and outliers was observed. Besides, the Spearman correlation test results indicated that the correlations coefficients among variables were lower than 0.90, and multicollinearity was not present. The results indicated that reading ability were related to parenting styles and HLE in children (Figure 2).

### 3.4 Association of dyslexia with HLE

**Figure 3:**
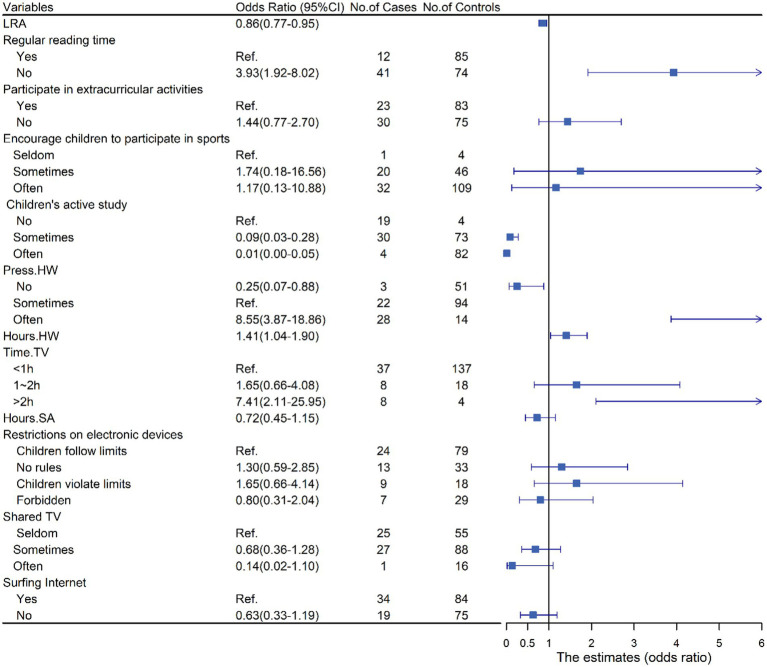
Associations (points) and 95% CI (bars) from Univariate logistic regression analyses of home literacy environment and dyslexia. Solid vertical lines indicate the null values. CI, confidence interval; LRA, literacy-related activities; Press. HW, Homework pressure; Hour. HW, hours of finishing homework; Time.TV, time spending on TV everyday; Hours.SA, hours of sports activities.

**Figure 4:**
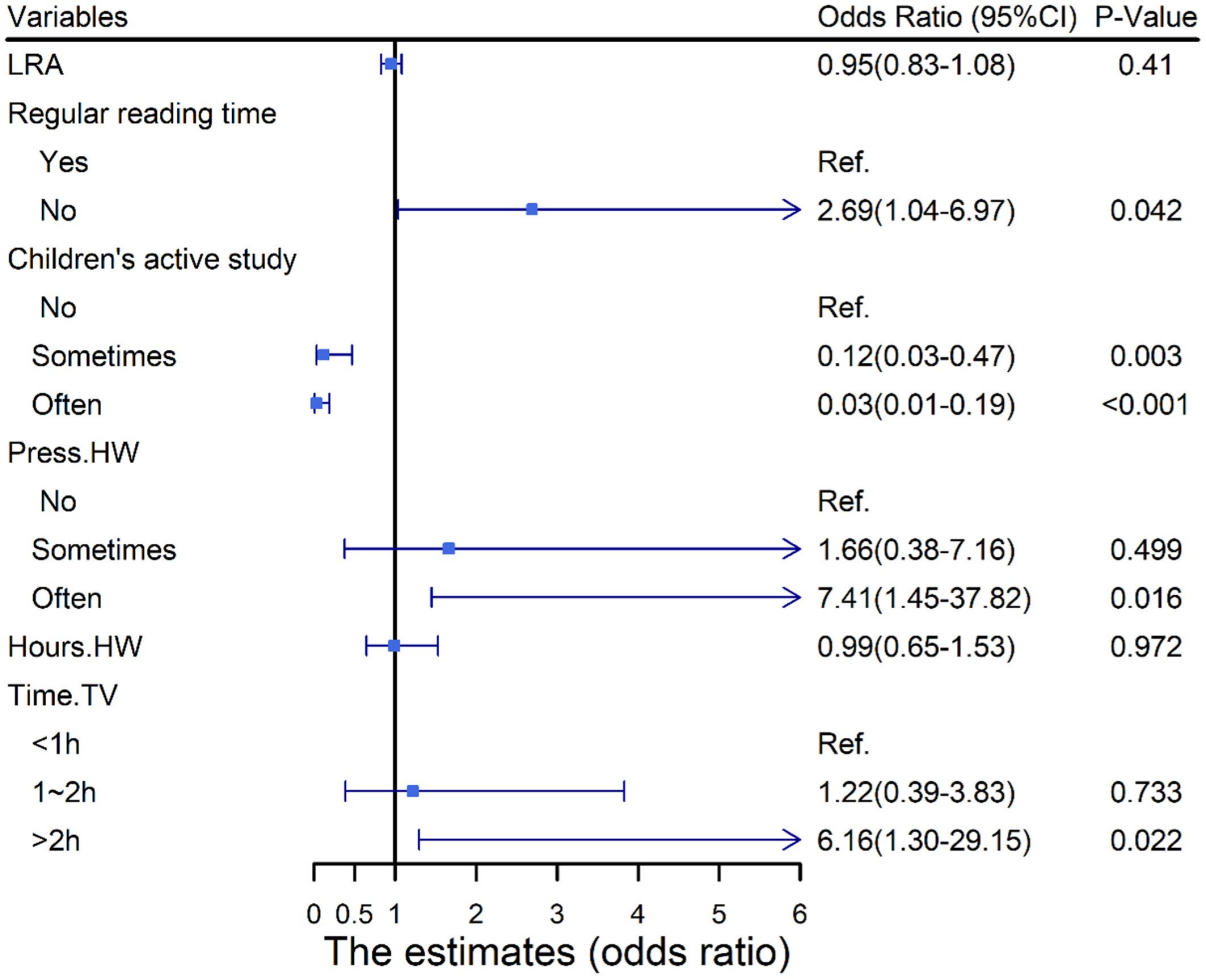
Associations (points) and 95% CI (bars) from multivariate logistic regression analyses of home literacy environment and dyslexia. Solid vertical lines indicate the null values. Adjusted for family income per month, be scolded by parents, ignore children’s feelings and family history of dyslexia. CI, confidence interval; LRA, literacy-related activities; Press.HW, Homework pressure; Hour.HW, hours of finishing homework; Time.TV, time spending on TV everyday.

In Figure 3, we showed the association between dyslexia and HLE. No regular reading time (OR = 3.93, 95%CI: 1.92–8.02, *p* < 0.05) and time spending on TV >2 h everyday (OR = 7.41, 95%CI: 2.11–25.95, *p* < 0.05) were positively associated with dyslexia. Moreover, the case group had higher homework pressure (OR = 8.55, 95%CI:3.87–18.86, *p* < 0.05) and longer homework time (OR = 1.41, 95% CI:1.04–1.90, *p* < 0.05) than the control group. While literacy-related activities (OR = 0.86, 95% CI: 0.77–0.95, *p* < 0.05), learning actively (*p* < 0.05) and absence of homework pressure (OR = 0.25, 95%CI: 0.07–0.88, *p* < 0.05) were protective factors for dyslexia. Likewise, we integrated the meaningful results into a multiple logistic regression model and demonstrated that dyslexia had a positive correlation with no regular reading time (OR = 2.69, 95%CI: 1.04–6.97), high homework pressure (OR = 7.41, 95%CI: 1.45–37.82), and time spending on TV >2 h everyday (OR = 6.16, 95%CI: 1.30–29.15, all *p* < 0.05). However, we did not observe any significant association between the risk of dyslexia and literacy-related activities and homework time (Figure 4).

### 3.5 Association between parenting styles and dyslexia

**Figure 5:**
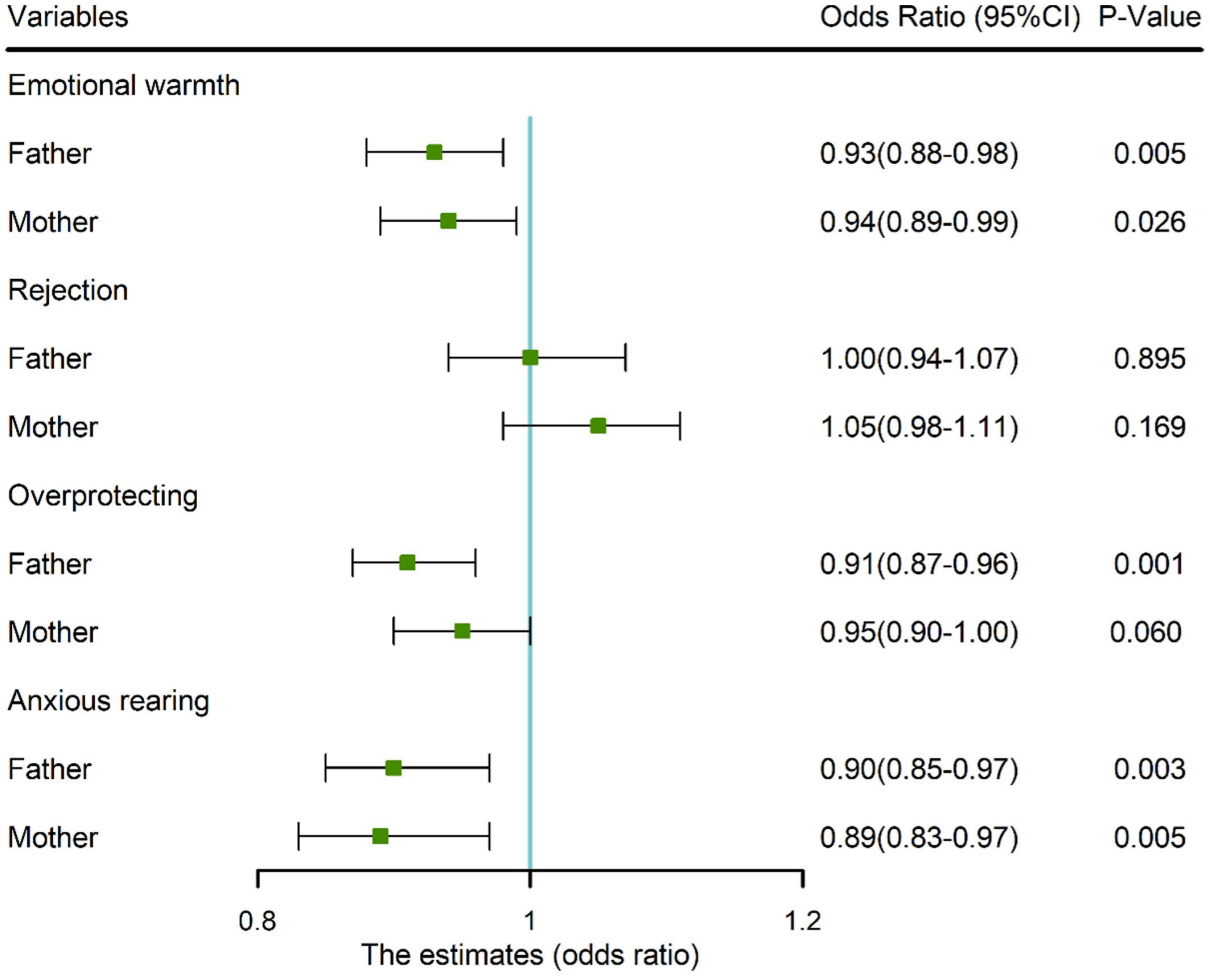
Associations (points) and 95% CI (bars) from multivariate logistic regression analyses of parental rearing style and dyslexia. Solid vertical lines indicate the null values. Adjusted for single parent family and place of residence. CI, confidence interval.

Similarly, we used the same regression model to identify predictors of parenting styles for dyslexia. We found that emotional warmth (Father: OR = 0.93, 95%CI: 0.88–0.98; Mother: OR = 0.94, 95%CI: 0.89–0.99), paternal overprotection (OR = 0.91, 95%CI: 0.87–0.96) and anxious rearing (Father: OR = 0.90, 95%CI: 0.85–0.97; Mother: OR = 0.89, 95%CI: 0.83–0.97) were negatively associated with dyslexia in children (all *p* < 0.05, Figure 5).

### 3.6 Test of the mediation model

**Figure 6:**
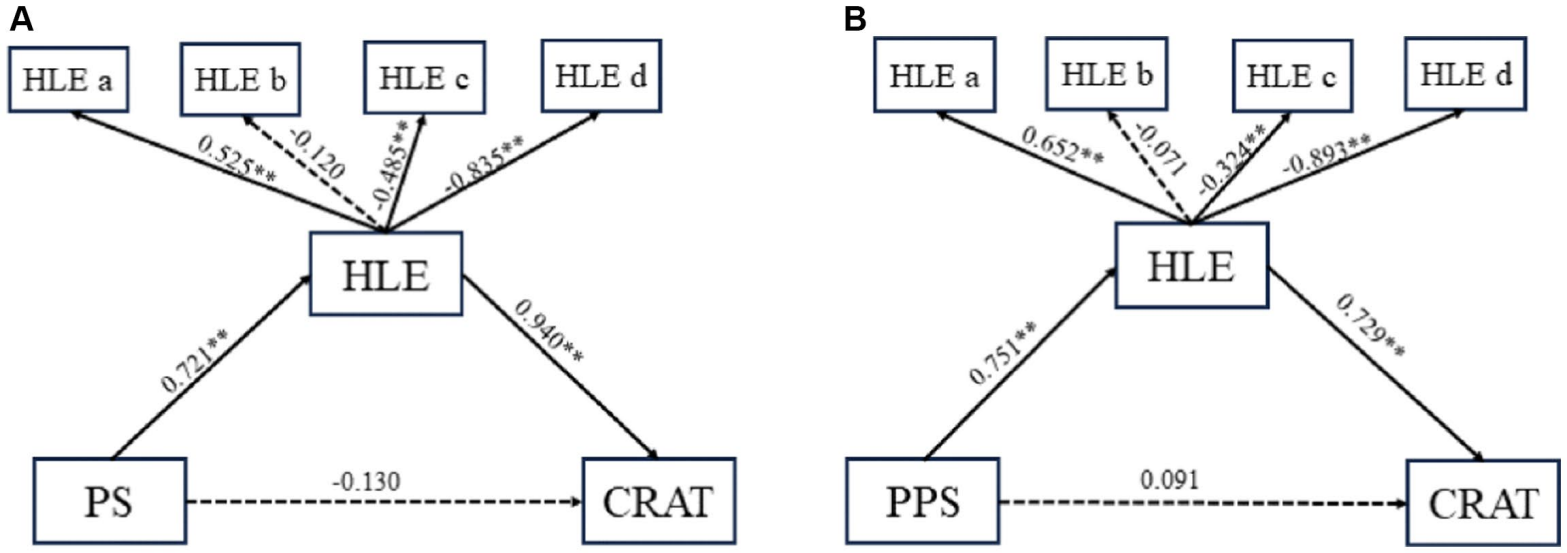
The chart and path coefficients of the mediators in the relationship between parenting styles and CRAT. **(A)** The mediation model of parenting styles on dyslexia. **(B)** The mediation model of paternal parenting styles on dyslexia. PS, parenting styles; PPS, paternal parenting style; HLE, home literacy environment; CRAT, the score of reading ability. *p < 0.05; **p < 0.01.

The results generated by M-plus version 8 were presented in Figure 6. The mediation analysis regarding parenting styles and dyslexia showed that after being adjusted for sex and age, parenting styles were positive predictors of HLE (β = 0.721, 95% CI: 0.448–0.852, *p* < 0.01). While the direct effect of parenting styles on dyslexia was not statistically significant (β = −0.130, 95% CI: −0.652 to 0.162, *p* > 0.05). Additionally, HLE had a positive effect on dyslexia (β = 0.940, 95% CI: 0.576–1.462, *p* < 0.01), which was divided into four components, namely HLEa, HLEb, HLEc, and HLEd (β = 0.525, −0.120, −0.485, −0.835). The mediation analysis results showed that HLE had a total mediation effect on the association between parenting styles and dyslexia. The total indirect effects of mediation were 0.55 and 0.68, respectively. The path model was a good fit (RMSEA = 0.096, CFI = 0.969, TLI = 0.924, SRMR = 0.039).

After conducting mediation effect analyses for both paternal and maternal parenting styles, taking into account factors of gender and age, the study found that both styles are associated with dyslexia (*p* < 0.01). However, the mediation model for maternal parenting styles did not reveal a mediating role for the HLE; in contrast, in the analysis for paternal parenting, the HLE did play a mediating role. Specifically, paternal parenting styles were positive predictors of HLE (β = 0.751, 95% CI: 0.536–0.854, *p* < 0.01), though their direct impact on dyslexia was insignificant (β = 0.091, 95% CI: −0.142-0.272, *p* > 0.05). Additionally, HLE had a positive effect on dyslexia (β = 0.729, 95% CI: 0.468–0.964, *p* < 0.01), which was divided into four parts, namely HLEa, HLEb, HLEc, and HLEd (β = 0.652, −0.071, −0.324, −0.893). The mediation analysis results showed that HLE had a total mediation effect on the association between paternal parenting styles and dyslexia. The total indirect effects of mediation were 0.64 and 0.55, respectively. The path model was a good fit (RMSEA = 0.114, CFI = 0.992, TLI = 0.985, SRMR = 0.042).

## 4 Discussion

Developmental dyslexia, a common learning disability affecting numerous children, has garnered increasing attention in recent years (Peterson and Pennington, 2012). Recent studies showed that children with dyslexia are at risk of academic failure and emotional problems (Yang et al., 2022). Therefore, investigating the factors that influence dyslexia may lead to mitigating the consequences of this prevalent disease. In the present study, we aim to examine the relationship between parenting styles and dyslexia among Chinese children, with a specific focus on the potential mediating role of HLE. Our findings indicate a strong correlation between dyslexia, HLE, and parenting styles. More importantly, HLE was found to have a complete mediation effect on the association between parenting styles and reading ability. This study builds upon previous research by introducing innovative models that connect parenting styles to dyslexia in children, thus offering valuable insights for future interventions and educational practices.

Our findings indicate that the absence of regular reading time and high homework pressure are risk factors for dyslexia. In line with previous research, our results highlight a significant association between dyslexia and HLE in children (Georgiou et al., 2021; Bigozzi et al., 2023). Ensuring children have a designated reading time not only increases their overall reading exposure but also cultivates positive reading habits, thereby enhancing their vocabulary and reading skills. A longitudinal study spanning ages 2–15 found that the HLE positively impacts language and literacy development in preschoolers, leading to improved reading comprehension throughout childhood due to preschool-based skills and heightened motivation (Torppa et al., 2022). Several studies have also suggested that the relationship between HLE and children’s reading ability may be reciprocal, with children’s early reading ability influencing parental involvement in home literacy activities (Silinskas et al., 2020; Georgiou et al., 2021). However, a study from Japan did not find such results, but rather linked access to literacy resources to early reading development (Tanji and Inoue, 2023). We posit that this disparity may be attributed to the temporal nature of HLE effects, which diminish as children receive formal literacy instruction. Nevertheless, it is indisputable that a favorable HLE offers numerous opportunities for educational activities that foster children’s language and literacy skills.

On the other hand, consistent with our findings, there were pieces of evidence showing that a conscious learning habit had a significant impact on the occurrence of dyslexia (Feng et al., 2022). Learning habit is an individual automatic learning behavior, conscious study habits are conducive to stimulate the enthusiasm and initiative of students to learn. Children with positive learning habits generally show positive learning attitudes in campus and home learning, and will actively and enthusiastically discuss with the teacher in the classroom or communicate with their classmates to learn after class, and express their views more actively, which greatly improves their language expression ability and expression opportunities. From this perspective, it can be explained that conscious and active learning habits can improve children’s reading experience, which has an important impact on the acquisition of children’s reading knowledge and skills, and thus reduces the risk of dyslexia (Romero-González et al., 2023). In contrast, the presence of longer time and stressful difficulties in completing homework assignments in dyslexic children, in addition to a possible lack of conscious study habits, is more likely to be due to their poor reading skills related to difficulties in completing homework tasks (Xiao et al., 2022).

In recent years, there has been a growing focus on parenting styles in the research community, yet little attention has been paid to the relationship between dyslexia and parenting styles (Kiuru et al., 2012; Huang et al., 2021). Similar to prior researches (Carotenuto et al., 2017; Zou et al., 2023), our research has found a strong correlation between parenting style and dyslexia. In particular, negative correlations were observed with regard to parental emotional warmth, anxious rearing, and fathers’ overprotection. These results may reflect apathetic and neglectful attitudes of parents toward dyslexic children and inadequate care for dyslexic children, which is a negative rearing style. Research has shown that parents who adopt a neglectful parenting style demand less from their children and communicate less with them (Otani et al., 2009). We suggest that negative parenting styles may be due to the fact that the academic underachievement of dyslexic children affects parents’ parenting satisfaction and sense of efficacy, which leaves most parents disillusioned and appearing less protective and anxious than parents of typically developing children (Chen, 2015). This may also partly explain our results. Consistent with our findings, a Finnish study found that parents of dyslexic children have high parenting stress and struggle to provide effective help (Natale et al., 2008). That is, more adaptive parenting styles promote a better performance when compared with less adaptive parenting styles. Previous studies have indicated that parenting styles play an important role in explaining higher reading processes (syntactic and semantic) in children with dyslexia, as supported by main theories on dyslexia (Carreteiro et al., 2016). Therefore, our results add to the growing body of evidence highlighting the impact of parenting styles on children with dyslexia.

In addition, we found that the relationship between boys’ parenting style and dyslexia was more pronounced. To our knowledge, there is a general consensus among current researchers that boys are at a higher risk of developing dyslexia than girls (Arnett et al., 2017; Feng et al., 2022; Yang et al., 2022). In line with a prior study, the gender of children had an impact on parenting styles (Zhong et al., 2023). Boys are given more responsibility, and parents are harsher, more rejecting and neglectful of boys, especially fathers, who are more likely to be strict with boys in home education. However, parents are more willing to tolerate girls. Also, there are differences in the personalities of boys and girls; boys are active and disobedient, while girls are more obedient and well-behaved, so parents are harsher and prone to punishment and rejection of boys, and are more tolerant and provide emotional warmth to girl (Barnett and Baruch, 1987). Thus, we should pay more attention to the way boys are parented, to be emotional warmth, tolerant and protective enough to help prevent dyslexia in boys.

Furthermore, our research has established that the HLE acts as an independent mediator between parenting styles and dyslexia. This means that positive parenting styles create a richer HLE, which in turn reduces the risk of dyslexia. Children’s early language skills are developed through their interactions with their parents (Glascoe and Leew, 2010). More specifically, parental attitudes toward reading seem to influence the development of children’s reading skills (Niklas et al., 2020). As numerous studies have shown, the actions and characteristics of both children and parents can shape family interactions (Shalani et al., 2023). Parents’ behaviors and attitudes, along with family communications and interactions, contribute to increasing children’s reading activities and create a positive HLE. The behavior of individual family members impacts all other members, making it essential to consider the family and the overall environment when examining a child’s behavior (Setyanisa et al., 2022). Given this, interventions that focus on effective parent–child communication can be a crucial part of rehabilitation training for children with dyslexia. Additionally, the mediation analysis revealed that the HLE fully mediates the relationship between paternal parenting styles and dyslexia in children. This may reflect a more pronounced interaction between fathers’ involvement in education and the HLE setting in fostering children’s reading skills. In contrast, no such mediation pattern emerged between maternal parenting styles and dyslexia, which might suggest differences in educational strategies or influences on the formation of children’s reading habits within the family culture. Moreover, this study will enhance our understanding of the impact of parenting styles on children’s dyslexia. In general, based on the findings of the present study, improving parenting styles, enhancing family interaction, and creating a positive family reading atmosphere can be considered crucial strategies for reducing the risk of dyslexia. In summary, parenting styles can either positively or negatively influence dyslexia in children through HLE, making this the most innovative contribution of this study.

Further to our conclusions, we propose that the complicated interaction observed between parenting styles, HLE, and dyslexia reveals patterns and influences that likely extend to a broader educational context. Given the universal importance of a supportive and enriching home literacy environment, these findings prompt a reconsideration of how we approach educational support for all students, particularly those facing any form of learning challenges (Leroux et al., 2024). The critical role of parenting and the home environment, as demonstrated in our study, suggests that similar mechanisms may influence educational outcomes across various learning difficulties, not limited to dyslexia (Şahin et al., 2020). Thus, integrating these findings, we advocate for the integration of parenting and home environment considerations into broader educational strategies and support systems, especially those students facing educational challenges.

This study has several limitations that must be considered. First, it is a case–control study, which limits the ability to establish causality. However, it provides valuable insights for future cohort or intervention studies seeking to verify these findings. Secondly, this study demonstrates that parenting styles impact children’s reading ability through the home literacy environment. However, defining the specifics of a “good” home literacy environment and establishing the optimal level to prevent reading difficulties and improve reading challenges remains elusive. Thirdly, the evaluation of parenting styles was subjective and may be subject to recall bias. Fourthly, the choice of control group from a single school may introduce selection bias. While the control group was chosen through random sampling that aims to minimize bias, selecting participants from a wider range of schools could further enhance the generalizability of our findings. Future research might benefit from a broader selection to verify and enhance the generalizability of the results. Additionally, factors such as parental occupation and educational level did not achieve statistical significance in our analyses. This finding may suggest that sample size, or the measures used to assess SES were not adequately sensitive to detect these effects. Therefore, future research should consider employing more comprehensive methodologies or larger samples to fully explore a broader range of potential mediators. To strengthen the generalizability of these findings, it is recommended to conduct future studies on samples from numerous regions to assess the reproducibility of these results.

## 5 Conclusion

In this study, we developed a robust mediation model to explore the potential relationship between parenting styles and dyslexia in children. Our findings suggest that the HLE serves as a mediator between parenting styles and dyslexia. This study highlights the critical role of parenting styles and the HLE in the development of interventions for dyslexic children. Our study offers fresh insights into the intricate connections between parenting styles and dyslexia, and the results carry significant theoretical and practical implications. This novel mediation model, never before utilized, aids in deepening our comprehension of the mechanisms that underlie the link between parenting styles and dyslexia. Additionally, the findings can inform the creation of clinical interventions and management programs aimed at reducing dyslexia occurrences among children. In conclusion, parenting styles appear to serve as a crucial factor in shaping individual differences in HLE and, ultimately, children’s reading abilities. Given that these parenting styles vary across different family backgrounds, they represent a promising target for interventions designed to enhance the HLE and support children’s reading development.

## Data availability statement

The raw data supporting the conclusions of this article will be made available by the authors, without undue reservation.

## Ethics statement

The studies involving humans were approved by the Ethics Committee of Mental Health Center, Shantou University Medical College. The studies were conducted in accordance with the local legislation and institutional requirements. Written informed consent for participation in this study was provided by the participants' legal guardians/next of kin.

## Author contributions

WW: Data curation, Formal analysis, Writing – original draft. XZ: Investigation, Methodology, Writing – review & editing. KW: Conceptualization, Supervision, Validation, Writing – review & editing. LG: Formal analysis, Visualization, Writing – review & editing. AH: Investigation, Methodology, Writing – review & editing. ZL: Data curation, Writing – review & editing. XY: Visualization, Writing – review & editing. QG: Data curation, Validation, Writing – review & editing. YH: Conceptualization, Funding acquisition, Project administration, Resources, Supervision, Validation, Writing – review & editing.
