# Supplementary material for: Association between parenting styles and dyslexia in primary school students: the mediating role of home literacy environment
Source: Front Psychol. 2024 Jun 13;15:1382519. doi: 10.3389/fpsyg.2024.1382519 (PMC11209994; doi:10.3389/fpsyg.2024.1382519)
Supplement: Supplementary file 1 [file Data_Sheet_1.docx]

**Association between parenting styles and dyslexia in primary school students: the mediating role of home literacy environment**

Table S1 Describe of home literacy environment variables.

Table S2 The result of the Chinese Reading Ability Test (CRAT) in the dyslexic group and the control group.

Table S1 Describe of home literacy environment variables.

| Variables | Items | Decoding of each response |
| --- | --- | --- |
| Literacy-related activities | The frequency of parents reading books to child since childhood | 1=never,2=occasionally,  3= often, 4 = always |
|  | Do parents encourage child to read books? | 1=never,2=occasionally,  3= often, 4 = always |
|  | Do parents buy the books which child was interested in? | 1=never,2=occasionally,  3= often, 4 = always |
|  | How often do parents buy new books for child? | 1 = per week or per month, 2 = per term, 3 = per year, 4 = buy when needed |
|  | The frequency of parents reading | 1=never,2=every day,  3= every week, 4 = every month |
|  | How much money do parents spend on books for child every year? | 1 = less than 150 CNY, 2 = 150 to 300 CNY, 3 =300 to 500 CNY, 4 = more than 500 CNY |
| Usage of electronic devices | Whether the child surf Internet | 1 = yes, 2 = no |
|  | Number of hours spent on TV every day | 1=less than 1 h , 2 = 1 to 2 h, 2 = 2 to 3 h, 3 = more than 3 h |
| Shared TV | Whether parents watching TV with children | 1 = seldom, 2 = sometimes, 3 = always |
| Restrictions on electronic devices | The restriction of time spending on TV/Internet at home? | 1=yes, children follow limits 2 = no rules, 3 = children violate limits, 4=No TV/Internet access |
|  | The parents’ attitude toward surfing internet? | 1=forbidden, 2 = Access to the Internet on holiday, 3 =sometimes ,4= allow |
| Children's learning habits | Do child have the habit of self-initiated learning? | 1 =often, 2 = occasionally, 3 = never |
| Completion of homework by children | The pressure of children finishing their homework | 1 = seldom, 2 = sometimes, 3 = always |
|  | Number of hours needed to finish homework (hours per day) |  |
|  | Whether child participates in extracurricular activity such as  reading? | 1=no,2= yes |
|  | Whether parents encourage children to participate activities? | 1 = seldom, 2 = sometimes, 3 = always |
|  | hours of outdoor |  |
|  | regular reading time | 1 = yes, 2 = no |

Table S2 The result of the Chinese Reading Ability Test (CRAT) in the dyslexic group and the control group.

| Variables | Dyslexic  (n=53) | Control  (n=159) | *t* | *P* |
| --- | --- | --- | --- | --- |
| Phonological awareness（$\bar{\text{x}}$±SD） |  |  |  |  |
| Tone scores | 8.26±3.46 | 10.16±2.97 | 3.569 | 0.001 |
| Onset scores | 8.13±2.41 | 9.31±1.91 | 3.641 | < 0.001 |
| Rime scores | 7.72±2.66 | 8.67±2.31 | 2.492 | 0.013 |
| Total scores | 24.11±6.36 | 28.14±5.73 | 4.308 | < 0.001 |
| Morphological awareness |  |  |  |  |
| Chinese word formation time(s) | 166.70±43.41 | 145.60±31.23 | 3.838 | < 0.001 |
| Chinese word formation scores | 9.40±0.88 | 9.81±0.58 | 3.148 | 0.002 |
| Rapid automatized naming |  |  |  |  |
| Time(s) | 18.91±4.30 | 14.11±2.58 | 7.670 | < 0.001 |
| Total scores | 2.18±0.42 | 2.92±0.57 | 10.141 | < 0.001 |
| Orthographic awareness |  |  |  |  |
| Non-character recognition scores | 15.57±1.91 | 16.37±1.63 | 2.988 | 0.003 |
| Radical position time(s) | 38.44±13.81 | 30.53±7.92 | 3.959 | < 0.001 |
| Radical position score | 10.23±1.83 | 10.96±1.26 | 2.725 | 0.008 |
| Reading ability |  |  |  |  |
| Number of words in 1min of reading | 190.91±35.96 | 218.71±43.83 | 4.172 | < 0.001 |
| Time for reading an article(s) | 95.66±21.53 | 86.02±30.55 | 2.127 | 0.035 |
| Total score of reading comprehension | 9.97±2.20 | 10.74±2.00 | 2.345 | 0.020 |
